# A Miniaturized Microwave Magnetometer with High Frequency Resolution Based on Diamond NV Centers for Multi-Microwave-Field Measurement

**DOI:** 10.3390/mi17060647

**Published:** 2026-05-25

**Authors:** Yaozhong Tian, Bo Wang, Qiang Zhu, Xin Li, Wenyuan Hao, Huanfei Wen, Jun Tang, Jun Liu

**Affiliations:** 1 State Key Laboratory of Extreme Environment Optoelectronic Dynamic Measurement Technology and Instrument, North University of China, Taiyuan 030051, China; tianyaozhong2023@163.com (Y.T.); wangbomail0913@163.com (B.W.); haowenyuannuc@163.com (W.H.); liuj@nuc.edu.cn (J.L.); 2 State Key Laboratory of Solid State Lighting, North University of China, Taiyuan 030051, China; tangjun@nuc.edu.cn; 3 State Key Laboratory of Science and Technology on Electronic Test and Measurement Laboratory, North University of China, Taiyuan 030051, China; wenhuanfei@nuc.edu.cn

**Keywords:** quantum sensor, NV center, microwave magnetic field, magnetometer

## Abstract

Diamond nitrogen-vacancy (NV) centers are regarded as promising microwave sensors owing to their excellent magnetic sensitivity, stability, and environmental compatibility. However, traditional confocal test platforms based on diamond NV centers are bulky, which limits their practical applications. In this paper, a fiber-coupled compact NV microwave magnetometer is designed that employs the continuous heterodyne measurement method and a fast Fourier transform to measure multiple microwave fields. We integrated the laser excitation module, microwave antenna module, and fluorescence collection module into a single unit, reducing the volume of the magnetometer to 13 cubic centimeters. By adjusting the frequency and power of the measured microwave signals, the applicability of the device under different frequency and power conditions was verified. Experimental tests show that the microwave magnetometer can simultaneously detect multiple microwave fields with different frequencies and power levels, achieving a frequency resolution on the order of millihertz (mHz) and a microwave detection sensitivity of 0.385 nT/Hz^1/2^. These results demonstrate the magnetometer’s multi-microwave-field measurement capability, making it highly promising for applications such as microwave anomaly localization and medical diagnosis.

## 1. Introduction

In scientific research and technological applications, near-field microwave detection technology has attracted extensive attention as a cutting-edge testing method [1,2]. By capturing and analyzing the microwave-frequency electrical parameter responses of samples, this technology enables precise measurement of the near-field microwave fields radiated from microwave integrated circuits and antennas [3,4,5]. At present, microwave devices are evolving rapidly toward miniaturization and integration, leading to a sharp increase in internal chip complexity and frequent electromagnetic interference. This trend imposes higher requirements on near-field microwave detection technology. Therefore, selecting an appropriate method for microwave field measurement is a crucial step to optimize the design of microwave devices and enhance their operational stability.

To meet the practical demands of near-field microwave detection, a variety of microwave detection instruments have been developed and deployed. Based on the metal probe microwave detection method, research teams have developed scanning microwave microscopes (SMMs) [6,7]. These devices offer nanoscale spatial resolution and operate by using a microwave probe to scan the sample surface, thereby acquiring electromagnetic characteristic information of the sample. However, this technology suffers from the drawback that the metal probe can introduce electromagnetic interference into the measured microwave field, thereby impairing the accuracy of weak-field measurements [8,9]. In the research direction of quantum microwave detection technology, scientists have developed superconducting quantum interference devices (SQUIDs) and Rydberg atom detection systems [10,11,12,13]. SQUIDs possess excellent temporal resolution, yet their operational scenarios are severely limited because they require cryogenic and vacuum environments [14]. Rydberg atoms exhibit ultra-high sensitivity to external electromagnetic fields and can be employed for high-precision measurements [15]. Nevertheless, the manipulation of Rydberg atoms demands extremely sophisticated technical means, and the Rydberg state is highly susceptible to interference from external environmental factors. In recent years, benefiting from the high sensitivity of diamond nitrogen-vacancy (NV) center sensing platforms to electromagnetic signals at room temperature, researchers have established diamond NV center sensing platforms on optical benches. The NV-center-based quantum sensing platform utilizes a diamond thin film containing NV centers as the readout probe for microwave field detection, and it boasts advantages such as a wide operating temperature range and fast detection speed [16,17,18]. However, such NV-center quantum sensing platforms built on optical benches are disadvantaged by their large size, which renders them inconvenient to move in practical applications and thus restricts their applicable scenarios.

Recently, various research groups have made substantial efforts to improve the performance of NV-center-based magnetometers for practical applications. For instance, integrating magnetic flux concentrators has been employed to enhance the effective magnetic field acting on the diamond [19]. Frequency-tracking schemes have been developed to extend the measurable dynamic range by continuously locking the microwave source to the shifting resonance peak [20]. Multi-channel microwave frequency modulation has enabled real-time vector magnetic field tracking by simultaneously addressing multiple NV axes [21]. Despite these advances, it can be observed that all these approaches focus on improving a specific local module (e.g., magnetic flux concentration, fluorescence collection, frequency locking, or multi-axis addressing). None of them directly address the capability of simultaneously detecting multiple microwave fields with different frequencies—a feature that is highly desirable for near-field microwave diagnosis and multi-source interference analysis. Therefore, there is an urgent need for a method that can perform multi-frequency microwave detection with high frequency resolution.

In this paper, we design a fiber-coupled compact NV microwave magnetometer. To achieve device portability, we have greatly simplified the optical path design by integrating the laser excitation module, microwave antenna module, and fluorescence collection module into a single unit, reducing the volume of the magnetometer to 13 cubic centimeters. Furthermore, a fiber-coupled laser is adopted to excite the NV centers, which significantly enhances the portability of the device. To verify the environmental adaptability of the device, systematic adjustments and experimental verifications of the frequency and power parameters of the measured microwave signals were conducted in this study. The results confirm that the microwave magnetometer exhibits stable applicability across a wide range of frequencies and powers. Experimental test results demonstrate that the device features multi-channel parallel detection capability, enabling accurate simultaneous detection of multiple microwave fields with different frequencies and power levels. Its frequency resolution can reach the millihertz (mHz) level, and the microwave detection sensitivity achieves 0.385 nT/Hz^1/2^, indicating excellent detection performance and great application potential.

## 2. Principles and Experimental

### 2.1. Experimental Principle

**Figure 1:**
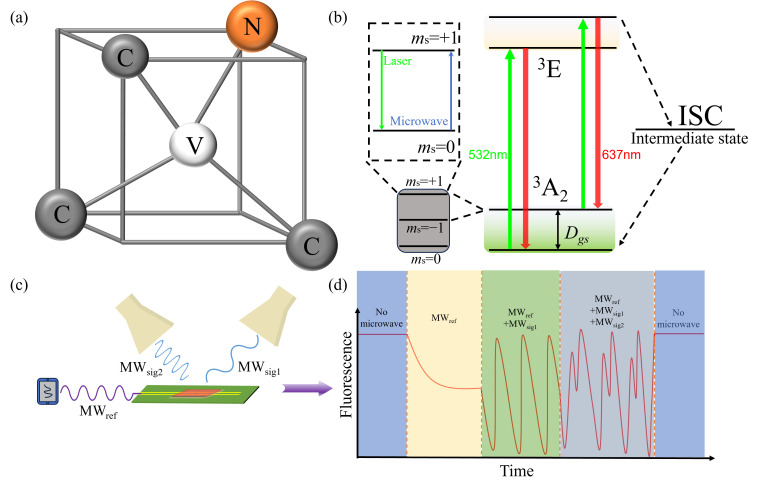
(**a**) Lattice structure of the NV center. (**b**) Energy level diagram for the NV center. (**c**) Schematic diagram illustrating the working principle of the microwave frequency detection system. (**d**) Schematic diagram showing the variation in the fluorescence signal when different signals act on the NV centers.

Nitrogen-vacancy (NV) centers are structural defects in diamond crystals, which are composed of a nitrogen atom (N) substituting a carbon atom in the lattice and an adjacent lattice vacancy (V), as illustrated in Figure 1a. Figure 1b depicts the energy level structure of NV centers, where both the ground state (^3^A_2_) and the excited state (^3^E) exhibit the characteristics of spin triplet states, consisting of three spin sublevels (|m_s_ = 0, ±1⟩) [22,23,24]. Through optical pumping with a 532 nm laser, electrons can be excited from the ground state to the excited state. Owing to the instability of the excited state, electrons return to the ground state via a radiative transition, accompanied by the emission of 637 nm red fluorescence. Due to the existence of a metastable relaxation channel inside, most electrons in the excited state |m_s_ = ±1⟩ undergo a non-radiative transition (ISC) through this metastable relaxation channel. Therefore, the fluorescence emission intensity of the |m_s_ = ±1⟩ state is significantly weaker than that of the |m_s_ = 0⟩ state. In the absence of an external magnetic field, the |m_s_ = ±1⟩ state shows degenerate characteristics, while there exists a zero-field splitting of 2.87 GHz between the spin sublevels of the ground state [25,26,27]. This feature enables coherent manipulation of quantum transitions between the spin sublevels of the ground state by microwave fields. Based on the significant difference in fluorescence intensity between different spin states, highly sensitive readout of the spin quantum states of NV centers can be achieved by real-time monitoring of the fluorescence signal.

In diamond NV centers, for a specific spin state |m_s_ = 0⟩, apart from the inherent longitudinal relaxation rate *Γ_1_*, an externally applied microwave field will open a transverse relaxation channel between |m_s_ = 0⟩ and |m_s_ = 1⟩. The relaxation rate *Γ_b_* of this channel can be expressed as [28]

(1) $$\Gamma_{b}=\frac{\gamma_{nv}^{2}b^{2}\Gamma_{2}}{2(\Gamma_{2}^{2}+\Delta^{2})}$$

where *γ_nv_* notes the gyromagnetic ratio of the electron spin in the NV center, b represents the amplitude of the microwave signal, *Γ_2_* denotes the transverse relaxation rate, and Δ represents the detuning rate between the microwave frequency and the resonant frequency of the NV center. The rate of polarizing the state ∣m_s_ = ±1⟩ to the state ∣m_s_ = 0⟩ is defined as *Γ_p_*. Under continuous light irradiation, *Γ_p_* competes with the relaxation processes.

When the competition reaches a steady-state equilibrium, the population *P_0_* of the state ∣m_s_ = 0⟩ can be expressed as

(2) $$P_{0}^{\infty }=\frac{1}{2}+\frac{\Gamma_{p}}{2(\Gamma_{p}+\Gamma_{1}+\Gamma_{b})}$$

The microwave signal induces the relaxation process represented by *Γ_b_*, which leads to the attenuation of the fluorescence signal. In the case of a weak microwave field where *Γ_b_* ≪ *Γ_1_*, the attenuation of the fluorescence signal ΔS can be expressed as [29]

(3) $$\Delta S\propto \Delta P_{0}^{\infty }\approx \frac{\Gamma_{p}\cdot \Gamma_{b}}{2(\Gamma_{p}+\Gamma_{1}{)}^{2}}\propto \Gamma_{b}\propto b^{2}$$

In the experiment, the synchronous identification of multi-signal frequencies is illustrated in Figure 1d. We simultaneously applied the reference signal *E_1_* and the signals under test *E_2_* and *E_3_* to the diamond NV centers. The reference signal and the signals under test can be expressed as

(4) $$\begin{array}{c} E_{1}=A_{1}\;\sin(\omega t+\phi_{1})\\ \begin{array}{c} E_{2}=A_{2}\;\sin((\omega +\delta_{1})t+\phi_{2})\\ E_{3}=A_{3}\;\sin((\omega +\delta_{2})t+\phi_{3}) \end{array} \end{array}$$

When two or more microwave fields in space undergo interference modulation, a heterodyne frequency is generated. As illustrated in Figure 1d, the fluorescence signal remains unchanged in the absence of an applied external microwave signal. After applying a reference microwave signal, the population of NV centers undergoes inversion, leading to a drop in the fluorescence signal. Upon further applying a microwave signal under test, the fluorescence signal oscillates in accordance with the frequency difference between the two microwave signals. On this basis, adding another microwave signal under test will cause the fluorescence signal to oscillate based on the sum and difference frequencies of all applied signals.

It is worth clarifying the applicability of the proposed heterodyne detection protocol to microwave signals with random phases. The beat signal generated between the reference microwave (*E_1_*) and a test signal (*E_2_*, with a frequency offset δ) can be expressed as a low-frequency cosine term: cos(δt+Δϕ), where *Δφ = φ_2_ − φ_1_* represents the initial phase difference. As shown in Equation (6), the phase difference only shifts the oscillation pattern in the time domain but does not affect the frequency component (δ) extracted by Fast Fourier transform (FFT). Therefore, as long as the test signal maintains a well-defined single frequency during the acquisition window (i.e., its frequency is stable, even if its absolute phase is unknown or randomly varying between measurements), the heterodyne frequency δ can be reliably detected. This holds true for both fully coherent microwave fields and those with random but constant phases over the FFT acquisition time. Only when the phase fluctuates rapidly within the acquisition window, causing spectral line broadening beyond the FFT resolution, would the frequency peak become less distinguishable—a scenario that is rare in near-field microwave device testing and is beyond the intended scope of this work.

### 2.2. Experimental Setup

**Figure 2:**
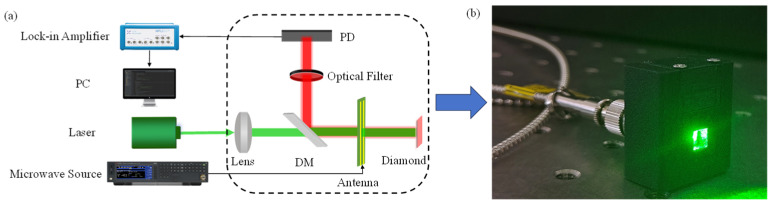
(**a**) Schematic diagram of the fiber-coupled magnetometer structure. (**b**) Photograph of the magnetometer prototype.

To improve the portability of the magnetometer and minimize the influence of external noise, a fiber-coupled laser was adopted to excite the NV centers, and miniaturized optical lenses were used to fabricate the magnetometer. The internal structure of the magnetometer is illustrated in Figure 2a. A green laser beam emitted by the 532 nm laser source (MGL-III-532, New Industrial Optoelectronics, changchun, China) with an output power of 80 mW was transmitted into the magnetometer via an optical fiber, and then collimated by a lens. Subsequently, the collimated laser beam passed through a dichroic mirror and a circular copper patch antenna (5 mm in diameter, made of 0.5 mm-diameter copper wire, placed approximately 1 mm above the diamond surface) to irradiate the diamond, thus polarizing the NV centers. In the experiment, the diamond synthesized by the high temperature and high pressure method (HPHT) with a size of 3 × 3 × 0.2 mm^3^ was cut along the [100] crystal direction, which effectively improved the lattice stability and prevented the accumulation of isotope impurities to avoid the influence of hyperfine splitting. After 2.5 h of electron irradiation (dose and energy were 9.8 × 10^15^ cm^−2^ and 10.0 MeV, respectively) and 2 h of annealing (850 °C), NV centers with a nitrogen concentration of 50 ppm were generated.

The red fluorescence signals generated by the NV centers were reflected by the dichroic mirror (OFD1LP-605B, JCOPTIX, Nanjing, China) into the fluorescence collection module. The fluorescence signals were filtered by an optical filter (EF10-600SP, LBTEK, Shenzhen, China) and then detected by a photodiode. The photodiode converted the fluorescence signals into electrical signals, which were fed into a lock-in amplifier (Zurich Instruments, HF2LI, Zurich, Switzerland). The lock-in amplifier converted the analog electrical signals into digital signals and transmitted them to a host computer. FFT was performed on the electrical signals using PyCharm 2021 software to extract the specific frequency information [30]. The photograph of the actual magnetometer prototype is shown in Figure 2b.

## 3. Results

### 3.1. Single Microwave Field Detection

First, the reference signal *E_1_* and the signal under test *E_2_* were applied to the NV centers. It can be observed that the red fluorescence emitted by the NV centers undergoes periodic variation at a specific frequency. This process can be expressed as

(5) $$E=E_{1}\times E_{2}=A_{1}\sin(\omega t+\phi_{1})\times A_{2}\sin((\omega +\delta_{1})t+\phi_{2})$$

Let *A_1_ = A_2_ = A* and $\phi_{1}$ = $\phi_{2}$ = ϕ. The above equation can be simplified as

(6) $$E=\frac{1}{2}\left[A\;\cos\left((2\omega +\delta_{1})t+\phi \right)+A\cos\left(\delta_{1}t\right)\right]$$

It can be concluded from the above equation that the NV centers generate two signals during the experiment. The frequency of the high-frequency signal corresponds to the sum of the frequencies of the two input signals, while that of the low-frequency signal equals the difference between them. Since the data acquisition device cannot capture signals with frequencies above 100 MHz, only the low-frequency signal is observable in the experiment.

The physical origin of the fluorescence modulation under multiple microwave fields can be understood as follows. The NV center’s spin population in the |m_s_ = 0⟩ state depends on the total microwave magnetic field experienced by the diamond. When two or more microwave fields (e.g., a reference field *E_1_* and test fields *E_2_*, *E_3_*, ⋯) coexist in space, the total field is the vector superposition, $E_{\mathrm{total}}=E_{1}+E_{2}+E_{3}+\cdots$. The spin transition rate $\Gamma_{b}$ is proportional to the square of the total microwave field amplitude, i.e., $I_{\mathrm{FL}}\propto |E_{\mathrm{total}}{|}^{2}$. Expanding the square generates cross terms such as $E_{1}\cdot E_{2}$, $\;E_{1}\cdot E_{3}$, and $E_{2}\cdot E_{3}$. Each cross term produces an oscillatory component at the difference frequency between the corresponding two fields. Because the photodetector and lock-in amplifier have a limited bandwidth (<100 MHz), only the low-frequency difference terms (typically <200 Hz) are retained, while high-frequency sum terms are filtered out. Consequently, the recorded fluorescence signal contains a superposition of beat notes at frequencies $\delta_{i}=|\omega_{i}-\omega_{\mathrm{ref}}|$, each corresponding to one test field. The amplitude of each beat note is proportional to the product of the amplitudes of the two interfering fields, enabling simultaneous extraction of both frequency and relative intensity of multiple microwave fields via FFT. This heterodyne principle underlies the multi-field detection capability of our magnetometer.

**Figure 3:**
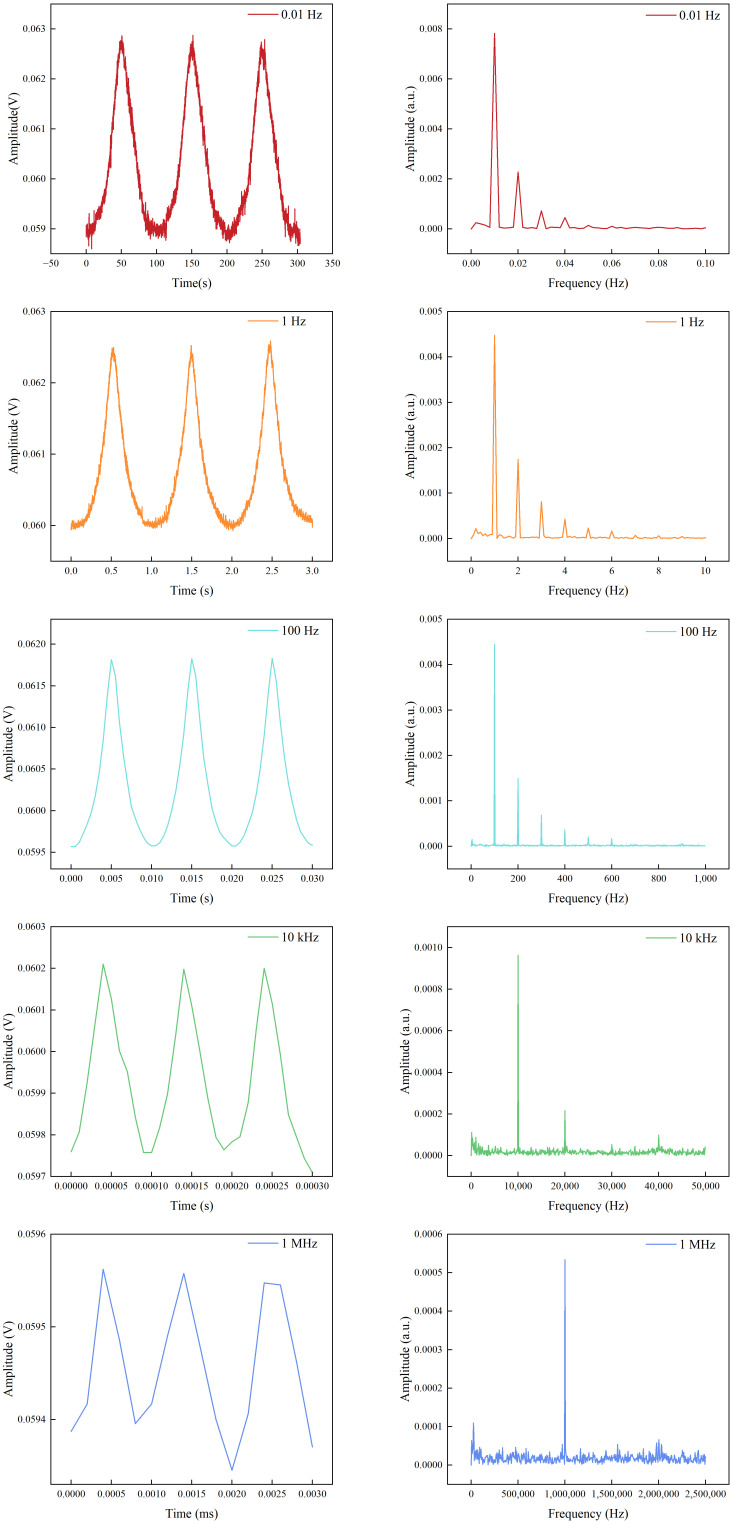
Variation in the fluorescence signal at different frequencies of the test signal *E_2_*.

To evaluate the frequency resolution and bandwidth of the system for single microwave frequency identification, the frequency and power of signal E_1_ were fixed at 2.86 GHz and 30 dBm, respectively. The power of signal *E_2_* was kept constant while its frequency was varied. Figure 3 illustrates the fluorescence variations at different frequency differences and presents the conversion of time-domain fluorescence data into frequency-domain information via FFT [31]. As shown in Figure 3, the minimum distinguishable frequency difference between *E_1_* and *E_2_* achieved by the system is 0.01 Hz, which is determined by the minimum frequency resolution of the microwave source employed in the experiment. Additionally, Figure 3 reveals that the amplitude of the fluorescence signal exhibits an overall decreasing trend with the gradual increase in the frequency difference. This phenomenon arises from the limited response rate of the fluorescence signal to changes in the microwave field, where a larger frequency difference leads to a more significant reduction in the fluorescence signal amplitude.

It should be noted that the frequency resolution of the heterodyne detection method is fundamentally limited by the signal acquisition time. According to the Fourier transform principle, the frequency resolution Δ*f = 1/T*, where T is the total measurement time. Therefore, achieving a higher resolution inevitably requires a longer acquisition time. In the experiment shown in Figure 3, the demonstrated frequency resolution of 0.01 Hz corresponds to an acquisition time of 100 s.

**Figure 4:**
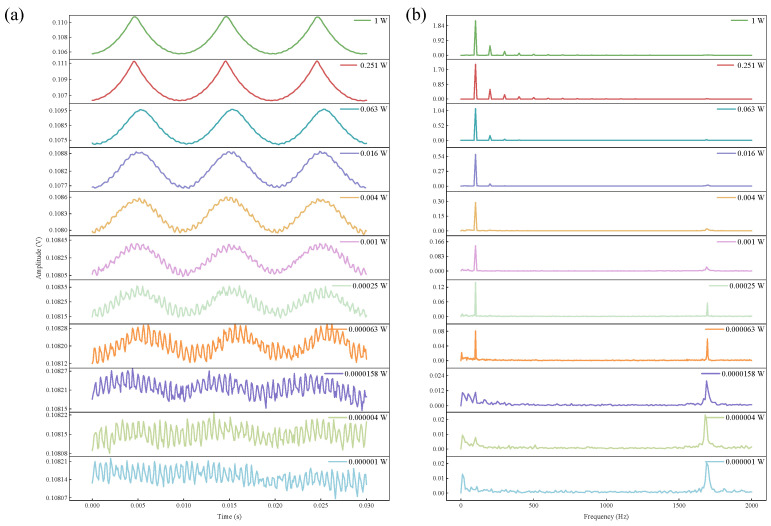
(**a**) Variation in the fluorescence signal at different power levels of the test signal *E_2_*. (**b**) Frequency-domain diagram of the fluorescence signal.

To measure the intensity of the microwave signal, the frequency difference between the reference signal *E_1_* and the test signal *E_2_* was fixed at 100 Hz, and the power of *E_2_* was adjusted accordingly. The measurement results are depicted in Figure 4. It can be observed that the amplitude of the fluorescence signal decreases progressively as the power of *E_2_* weakens. This observation is attributed to the reduced microwave intensity exerted on the diamond NV centers caused by the decreasing power of the test signal.

It should be noted that when the microwave signal power is low, weak oscillatory components appear at frequencies above 1.5 kHz. These components originate from the intrinsic noise floor of the measurement system, including the residual reference frequency and harmonics of the lock-in amplifier, as well as broadband electronic noise from the photodetector and data acquisition electronics. Under high microwave power, the heterodyne signal at the difference frequency is strong enough to dominate the power spectral density, effectively masking these high-frequency noise peaks. When the microwave power decreases, the heterodyne amplitude decreases proportionally, making the otherwise negligible system noise relatively visible. These high-frequency oscillations do not affect the extraction of the low-frequency heterodyne signal.

**Figure 5:**
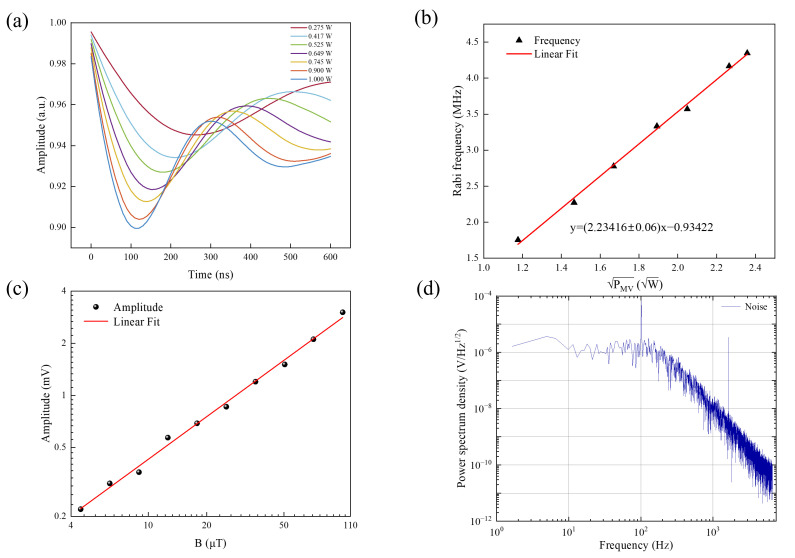
(**a**) Rabi oscillations at different power levels. (**b**) Linear relationship between different power levels and Rabi frequency. (**c**) Relationship between different microwave intensities and fluorescence signal amplitudes. (**d**) Power spectral density of the fluorescence signal.

The microwave is radiated from the antenna with a field strength of $b=k\sqrt{P_{MW}}$, where P_MW_ denotes the input microwave power. When the microwave field is sufficiently strong, the time trace of the fluorescence signal exhibits Rabi oscillations [32,33]. The measured Rabi frequencies under different microwave power levels are illustrated in Figure 5a. The Rabi frequency $\Omega =\gamma_{NV}b/\sqrt{2}$ shows a linear relationship with $\sqrt{P_{MW}}$, and the derived relational expression is presented in Figure 5b. As depicted in Figure 5c, the amplitude of the measured fluorescence signal exhibits a one-to-one correspondence with the microwave intensity, yielding the expected linear response. This verifies the effectiveness of the proposed method in microwave field strength detection, *k* = 0.818. Subsequently, the amplitude spectral density (ASD) of the magnetometer was measured using a lock-in amplifier over a duration of 100 s, and the voltage noise spectral density $S_{V}$ was averaged in the frequency range of 1–100 Hz (excluding the 50 Hz power-line harmonic), as shown in Figure 5d. The sensitivity of the magnetometer is then determined as $\eta =S_{V}/k=0.385\;\mathrm{nT}/\sqrt{Hz}$, based on the ASD and the linear relationship between the fluorescence signal amplitude and the microwave intensity.

A practical concern regarding the detection scheme is whether the reference microwave signal *E_1_* is always necessary and whether such a high-power microwave background could hinder the detection of weak test fields. The reference field is indeed indispensable for the heterodyne process; without it, the test signal alone would only cause a static reduction in the fluorescence (Figure 1d), yielding no oscillatory component from which the frequency could be extracted via FFT. As for potential interference, the high-power reference operates at a Rabi frequency much higher than the heterodyne difference frequency (*δ*~0.01–200 Hz). Its effect on the time-trace is essentially a constant offset that does not modulate the beat note amplitude. Moreover, the lock-in amplifier and subsequent FFT analysis focus exclusively on the low-frequency oscillatory component (<200 Hz), effectively rejecting any static or high-frequency contributions from the reference field. This is experimentally confirmed by the fact that weak test signals (down to −20 dBm in Figure 4) remain clearly distinguishable even in the presence of the 30 dBm reference. Therefore, the strong reference does not compromise the sensitivity or reliability of weak microwave field detection.

### 3.2. Multi-Microwave-Field Measurement

**Figure 6:**
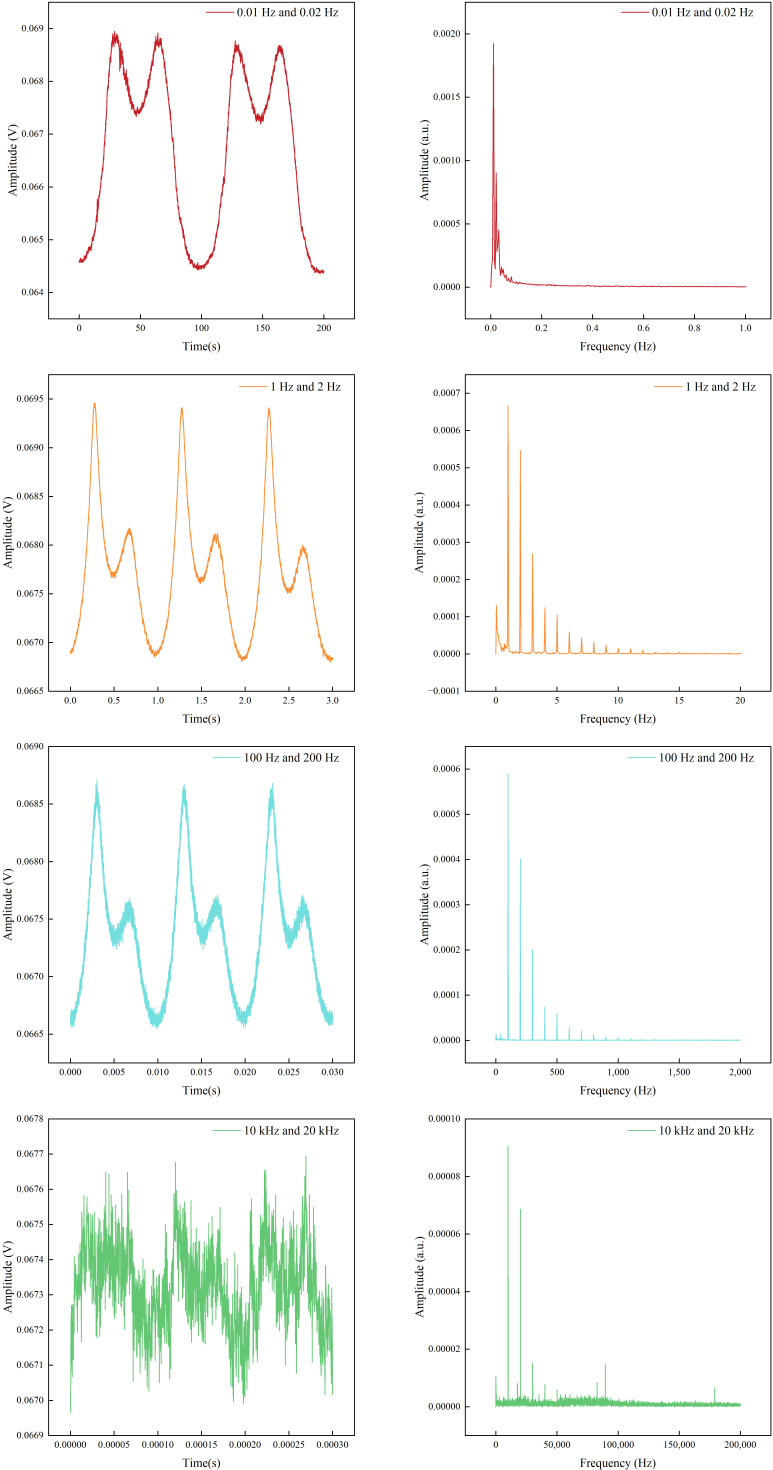
Variation in the fluorescence signal at different frequencies of the test signals *E_2_* and *E_3_*.

To expand the application scope of the magnetometer, a new test signal *E_3_* was introduced into the system. The power and frequency of the reference signal *E_1_* were kept constant, as were the power levels of the test signals *E_2_* and *E_3_*. The measurement results obtained by varying the frequencies of the test signals are illustrated in Figure 6. It can be observed that as the frequencies of the test signals *E_2_* and *E_3_* increase gradually, the amplitude of the fluorescence signal decreases progressively, and the fluorescence signal becomes disordered. This phenomenon arises because the increasing frequencies of the test signals lead to the introduction of other high-frequency microwave signals, increasing the noise in the collected fluorescence signal.

**Figure 7:**
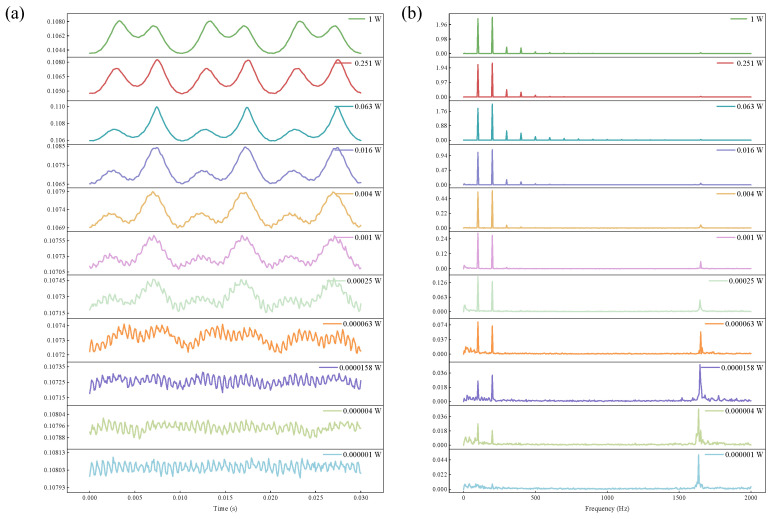
(**a**) Variation in the fluorescence signal at different power levels of the test signals *E_2_* and *E_3_*. (**b**) Frequency-domain diagram of the fluorescence signal.

Subsequently, the frequencies of the test signals *E_2_* and *E_3_* were fixed, while their power levels were adjusted. The corresponding measurement results are presented in Figure 7. It is evident that with the synchronous decrease in the power of *E_2_* and *E_3_*, the amplitude of the fluorescence signal declines gradually, and the noise contained in the fluorescence signal becomes increasingly prominent. When the power decreases to 0.004, the fluorescence signal cannot be observed in the time domain due to the interference of noise. Nevertheless, a weak signal can still be detected in the frequency domain after FFT processing.

Similar high-frequency noise features as discussed in Section 3.1 become visible in Figure 7b when the test signals are weak, for the same reason that the heterodyne signal can no longer mask the system noise floor.

To quantitatively evaluate the multi-microwave-field detection performance, we measured the frequency accuracy, amplitude error, crosstalk, and detection limit under simultaneous three-field operation (reference *E_1_* plus test signals *E_2_* and *E_3_* at beat frequencies of 50 Hz and 80 Hz). The frequency accuracy was better than 0.01 Hz, the amplitude error below 2%, the crosstalk below −35 dB, and the detection limit (SNR = 3) around 1.20 nT, demonstrating reliable and quantitative multi-field detection capability.

To demonstrate the performance advantages of the proposed microwave magnetometer in multi-field measurement, we compared the performance of various mainstream technical approaches, with detailed data summarized in Table 1. Most existing studies focus on improving sensitivity or dynamic range through methods such as magnetic flux concentration, frequency tracking, or multi-channel vector demodulation. In this work, while maintaining a competitive sensitivity of 0.385 nT/Hz^1/2^, we achieve millihertz-level frequency resolution and simultaneous multi-microwave-field detection. This provides a universal solution for near-field microwave diagnosis, multi-source interference analysis, and frequency-response characterization of microwave devices.

## 4. Conclusions

The measurement technology for the frequency and intensity of microwave signals constitutes an important technical means for identifying the operating status of microwave devices. In this work, we designed a fiber-coupled miniaturized microwave magnetometer, which employs the continuous heterodyne measurement method and FFT to realize the detection of multiple microwave fields. The volume of the magnetometer was reduced to 13 cubic centimeters, which significantly enhances its portability. By conducting measurements on the microwave signals under test with different frequencies and power levels, the applicability of the device under varying frequency and power conditions was verified. Experimental tests demonstrate that the proposed microwave magnetometer can simultaneously detect multiple microwave fields with different frequencies and power levels, achieving a frequency resolution on the order of millihertz (mHz) and a microwave detection sensitivity of 0.385nT/Hz^1/2^. These results indicate that the designed integrated NV magnetometer exhibits excellent performance in microwave field detection and holds broad application prospects.

In future work, we will adopt micro/nano fabrication processes and structures to further reduce the size of the magnetometer. In addition, external devices, such as microwave sources, lock-in amplifiers, and host computers, will be redesigned to further improve the portability of the system. Meanwhile, a microwave focusing structure will be incorporated. By virtue of the amplification effect of the microwave focusing structure on microwave fields, the interference of environmental noise will be minimized, and the sensitivity of the magnetometer will be further enhanced.

## Figures and Tables

**Table 1:** Comparison Table of Performance Parameters of Different Microwave Magnetometers

Groups	Sensitivity (nT/Hz^1/2^)	Volume (cm^3^)	Function
Fu et al. [21]	0.93	Portable	Real-Time Vector Magnetic Measurement
Wang et al. [20]	3.58	~12	High Dynamic Range Magnetic Field Measurement
Mao et al. [19]	0.34	3.81	High-Sensitivity Magnetic Field Detection
Our work	0.385	13	Multi-Frequency Microwave Magnetic Field Detection

## Data Availability

Data underlying the results presented in this paper are not publicly available at this time. However, they may be obtained from the authors upon reasonable request.
